# RSK signaling in myeloid malignancies: biology, disease dependency, and therapeutic targeting

**DOI:** 10.3389/fcell.2026.1900741

**Published:** 2026-07-10

**Authors:** Gavin M. Traber, Sandra E. Dunn, Kathleen M. Sakamoto

**Affiliations:** 1 Division of Hematology/Oncology, Department of Pediatrics, Stanford University School of Medicine, Stanford, CA, United States; 2 Phoenix Molecular Designs, Vancouver, BC, Canada

**Keywords:** acute myeloid leukemia, myeloproliferative neoplasms, ribosomal S6 kinase, signal transduction, targeted therapy

## Abstract

Acute myeloid leukemia (AML) and related myeloid malignancies remain clinically challenging despite recent advances in molecular profiling and targeted therapy. Although recurrent oncogenic drivers have improved disease classification and informed therapeutic development, durable disease control remains limited by signaling plasticity and therapeutic resistance of heterogeneous malignant stem and progenitor populations. These limitations drive continued interest in signaling dependencies that investigate oncogenic inputs across genetically diverse myeloid disease states. The p90 ribosomal S6 kinase (RSK) family has emerged as one such candidate. Positioned as a downstream convergence of MAPK/ERK and PI3K/PDK-1 signaling, RSK regulates proliferation, survival, translational control, inflammatory signaling, and cellular stress adaptation, all processes directly relevant to malignant progression. Growing evidence supports aberrant RSK activation in AML, functional dependency in FLT3-mutant AML and myeloproliferative neoplasms/myelofibrosis, and increasing translational interest in selective pharmacologic inhibition. Early preclinical studies demonstrate anti-leukemic activity through both genetic and pharmacologic targeting, while emerging clinical development of RSK inhibitors in solid tumors supports therapeutic feasibility. Here, we review the biologic role of RSK signaling in myeloid malignancies and discuss the therapeutic opportunities and challenges associated with targeting this signaling axis.

## 1 Introduction

Myeloid malignancies comprise a biologically heterogeneous group of hematologic neoplasms defined by disrupted differentiation, aberrant proliferation, altered survival signaling, and progressive failure of normal hematopoiesis (De Kouchkovsky and Abdul-Hay, 2016; Papaemmanuil et al., 2016; Newell and Cook, 2021; Dohner et al., 2022). This disease spectrum includes acute myeloid leukemia (AML), myeloproliferative neoplasms (MPNs), myelodysplastic syndromes (MDS), myelofibrosis (MF) and secondary leukemic transformation that emerge through the accumulation of genetic, epigenetic, and signaling abnormalities that reshape hematopoietic stem and progenitor cell behavior (De Kouchkovsky and Abdul-Hay, 2016; Papaemmanuil et al., 2016; Newell and Cook, 2021; Dohner et al., 2022; Kong et al., 2025). Despite major advances in molecular classification and targeted therapy, durable disease control remains difficult in aggressive, relapsed, or biologically adaptive myeloid disease states (Papaemmanuil et al., 2016; Daver et al., 2019; Kazi and Ronnstrand, 2019; Dohner et al., 2022; Koschade et al., 2022).

AML remains a clinically relevant acute myeloid malignancy that presents substantial therapeutic challenges (De Kouchkovsky and Abdul-Hay, 2016; Papaemmanuil et al., 2016; Newell and Cook, 2021; Dohner et al., 2022). AML is clinically characterized by clonal expansion of immature myeloid blasts within the bone marrow, peripheral blood, and extramedullary tissues (De Kouchkovsky and Abdul-Hay, 2016; Newell and Cook, 2021). If left untreated, AML may result in suppression of normal hematopoiesis, cytopenias, immune dysfunction, and ultimately organ failure (De Kouchkovsky and Abdul-Hay, 2016; Newell and Cook, 2021). Large-scale sequencing efforts have defined AML as a molecularly heterogeneous disease shaped by recurrent alterations affecting receptor tyrosine kinases, transcription factors, chromatin modifiers, spliceosome machinery, tumor suppressors, and metabolic regulators (Papaemmanuil et al., 2016; Dohner et al., 2022). Among these, activating FMS-like tyrosine kinase 3 (FLT3) alterations remain one of the most clinically actionable molecular subsets, occurring in approximately 15%–30% of AML cases and consistently associating with aggressive disease biology, increased relapse risk, and inferior survival outcomes (Daver et al., 2019; Kazi and Ronnstrand, 2019).

FLT3 inhibitor development represented a major advance in genotype-directed AML therapy, demonstrating that targeting recurrent oncogenic drivers can improve clinical response rates (Daver et al., 2019; Kazi and Ronnstrand, 2019), yet durable disease control remains limited. Clinical benefit may also be constrained by treatment-related toxicities, including cytopenias such as neutropenia, QT interval prolongation, edema, and gastrointestinal adverse effects (Daver et al., 2019). Resistance emerges through kinase domain mutation, adaptive pathway rewiring, compensatory signaling activation, altered apoptotic priming, microenvironment-mediated protection, and persistence of leukemic stem and progenitor populations that evade therapeutic eradication (Koschade et al., 2022). In many cases, malignant cells do not need to restore the initiating oncogenic lesion if downstream survival and proliferative signaling remain intact. This has shifted therapeutic interest toward targeting convergent signaling dependencies that sit downstream of genetically diverse drivers.

The same logic applies beyond AML. Chronic forms of myeloid malignancies such as MPNs are sustained by persistent oncogenic signaling through JAK-STAT, MAPK, and PI3K-associated pathways downstream of canonical driver mutations in JAK2, CALR, and MPL signaling proteins (Vainchenker and Kralovics, 2017; Bose and Verstovsek, 2020). Although JAK inhibitor therapy improves symptom burden and splenomegaly, molecular eradication remains uncommon, and progression to accelerated- or blast-phase disease remains a major clinical challenge (Bose and Verstovsek, 2020). Across myeloid malignancies, malignant cells repeatedly exploit redundant signaling architecture, adaptive stress responses, and stem-like persistence programs that limit the long-term efficacy of therapies directed exclusively at upstream lesions.

Within this framework, signaling effectors that integrate multiple oncogenic inputs are increasingly attractive therapeutic candidates. The p90 ribosomal S6 kinase (RSK) family is one such example. RSK proteins function downstream of MAPK/ERK and PI3K/PDK-1 signaling whereby it regulates cell-cycle progression, translational control, apoptosis resistance, inflammatory signaling, stress adaptation, and lineage-associated transcriptional programs (Romeo et al., 2012; Youn et al., 2021; Kong et al., 2024; Kong et al., 2025; Traber et al., 2026). These outputs are directly relevant to leukemogenesis, disease progression, and therapeutic resistance.

RSK is increasingly viewed as more than a passive downstream kinase. In fact, elevated RSK activation has been identified in primary AML patient samples and associates with inferior clinical outcomes (Chae et al., 2020). Functional studies support disease dependency through both pharmacologic and genetic targeting approaches in AML, FLT3-mutant disease, and MPN-associated transformation (Chae et al., 2020; Youn et al., 2021; Kong et al., 2024; Kong et al., 2025; Traber et al., 2026). Recent translational work also suggests selective RSK inhibition may suppress malignant progression while preserving a meaningful degree of normal hematopoietic function, raising the possibility of a clinically useful therapeutic window (Traber et al., 2026). In this review, we examine the biologic role of RSK signaling in myeloid malignancies and discuss the opportunities and challenges associated with therapeutic targeting of this signaling axis.

## 2 Biology of RSK signaling in hematologic malignancy

**FIGURE 1:**
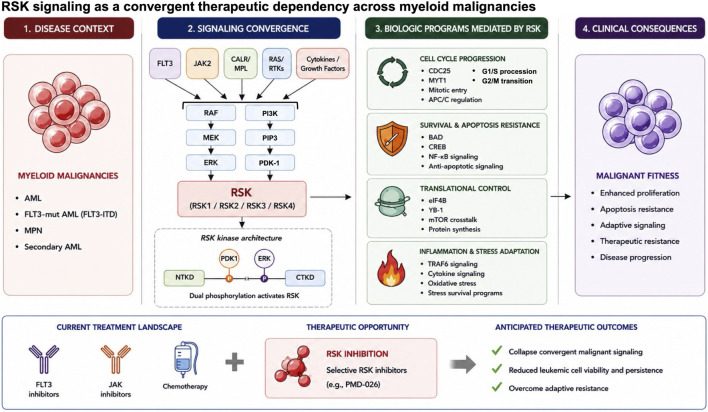
RSK signaling as a convergent therapeutic dependency and therapeutic vulnerability in myeloid malignancies. Convergent signaling architecture across myeloid malignancies, including acute myeloid leukemia (AML), FLT3-mutant AML, myeloproliferative neoplasms (MPNs), and secondary AML, illustrating integration of diverse oncogenic inputs through the MAPK/ERK/PDK-1-RSK signaling axis. Activated RSK regulates multiple downstream biologic programs, including cell-cycle progression, survival and apoptosis resistance, translational control, inflammatory signaling, and stress adaptation, which collectively contribute to malignant cell fitness, therapeutic resistance, and disease progression. Because RSK functions as a common downstream signaling effector, selective RSK inhibition may provide an opportunity to disrupt shared malignant signaling outputs across genetically diverse myeloid malignancies while complementing existing therapeutic strategies.

The RSK family consists of four serine/threonine kinase isoforms: RSK1 (*RPS6KA1*), RSK2 (*RPS6KA3*), RSK3 (*RPS6KA2*), and RSK4 (*RPS6KA6*) (Romeo et al., 2012; Youn et al., 2021; Kong et al., 2024; Kong et al., 2025; Traber et al., 2026). These kinases function as downstream effectors of the canonical RAS-RAF-MEK-ERK and PI3K/PDK-1 signaling cascade, linking extracellular growth factor and cytokine signaling to transcriptional and post-translational cellular responses (Romeo et al., 2012; Youn et al., 2021; Kong et al., 2024; Kong et al., 2025) (Figure 1). While structurally related, RSK isoforms demonstrate tissue-specific expression patterns and increasingly appear to exhibit context-dependent biologic functions rather than complete redundancy (Romeo et al., 2012; Youn et al., 2021; Kong et al., 2024; Kong et al., 2025; Traber et al., 2026).

RSK proteins are structurally distinct from many conventional kinases because they contain two functional kinase domains within their polypeptide sequence (Romeo et al., 2012; Youn et al., 2021). The C-terminal kinase domain (CTKD) participates in activation, while the N-terminal kinase domain (NTKD) serves as the primary substrate-phosphorylating catalytic domain (Romeo et al., 2012; Youn et al., 2021). Canonical activation begins following extracellular stimulation that activates ERK1/2. Activated ERK phosphorylates the CTKD, initiating sequential phosphorylation and autophosphorylation events that recruit phosphoinositide-dependent kinase 1 (PDK1), ultimately activating the NTKD and enabling downstream substrate phosphorylation (Romeo et al., 2012; Youn et al., 2021). This convergence is particularly relevant in myeloid malignancies, where genetically distinct upstream oncogenic drivers can funnel into shared downstream signaling dependencies centered on RSK (Figure 1).

RSK also contributes substantially to survival signaling (Romeo et al., 2012; Beeram et al., 2021; Youn et al., 2021; Traber et al., 2026). Multiple studies have identified RSK-mediated phosphorylation of regulators involved in apoptosis resistance and pro-survival signaling, including BAD, CREB, and NF-κB-associated pathways (Romeo et al., 2012; Beeram et al., 2021; Youn et al., 2021; Traber et al., 2026). Through these interactions, RSK suppresses apoptotic signaling and reinforces malignant cell fitness under proliferative, metabolic, and therapeutic stress. Moreover, RSK regulates multiple phases of cell-cycle progression through phosphorylation of proteins involved in G1 progression, G2/M transition, mitotic entry, and spindle checkpoint control, including CDC25 phosphatases, MYT1-associated signaling proteins, and other cell-cycle regulatory machinery (Romeo et al., 2012; Youn et al., 2021). Consistent with these functions, pharmacologic RSK inhibition in AML models disrupts cell-cycle progression, resulting in G2/M accumulation and impaired mitotic exit (Chae et al., 2020), while more recent studies using PMD-026 further support RSK inhibition as a functional therapeutic vulnerability in AML (Kong et al., 2024; Kong et al., 2025).

RSK additionally participates in translational regulation, an increasingly important area in malignant hematopoiesis (Romeo et al., 2012; Chae et al., 2018; Youn et al., 2021; Kong et al., 2024) (Figure 1). RSK signaling intersects with mTOR-associated protein synthesis pathways and regulates translational effectors including eIF4B and YB-1 (Romeo et al., 2012; Youn et al., 2021; Perner et al., 2022). YB-1 supports translation of oncogenic transcripts linked to malignant fitness and therapeutic persistence (Perner et al., 2022). Inflammatory and stress-adaptive signaling represent another important layer of RSK biology (Romeo et al., 2012; Yao et al., 2018); Figure 1). Studies have implicated RSK in cytokine-responsive signaling, NF-κB activation, oxidative stress adaptation, and inflammatory pathway reinforcement, including TRAF6-associated signaling (Romeo et al., 2012; Yao et al., 2018).

Historically, RSK isoforms were often discussed as broadly redundant downstream MAPK and PI3K effectors. Much of this mechanistic understanding emerged through pharmacologic interrogation using early tool inhibitors with differing isoform selectivity and kinase domain targeting, although these compounds were not designed for clinical translation (Table 1). That view has become less convincing as functional dependency studies continue to develop. Earlier mechanistic studies implicated RSK2 in FLT3-driven leukemic signaling, while more recent work increasingly identifies RSK1 as a dominant vulnerability in AML and MPN contexts (Yao et al., 2018; Kong et al., 2024; Kong et al., 2025; Traber et al., 2026). Whether these observations reflect isoform specialization or disease-context dependence remains unresolved.

**TABLE 1:** Representative pharmacologic RSK inhibitors and translational development status.

Inhibitor	Chemical class/Binding mode	Primary RSK target	Key findings	Development status	Representative references
SL0101	Natural product-derived kaempferol glycoside; reversible inhibitor	NTKD; relative selectivity for RSK1/2	Established early proof-of-concept for selective pharmacologic RSK inhibition; limited by modest potency and stability	Preclinical research tool	Smith et al. (2005), Youn et al. (2021)
FMK	Covalent irreversible small molecule inhibitor	CTKD; predominantly RSK1/2/4	Mechanistic probe demonstrating irreversible RSK inhibition; primarily useful for pathway interrogation	Preclinical research tool	Cohen et al. (2005), Romeo et al. (2012)
LJH685	ATP-competitive selective small molecule	NTKD; pan-RSK	Improved potency/selectivity versus first-generation compounds; widely used in mechanistic studies	Preclinical research tool	Aronchik et al. (2014)
LJI308	ATP-competitive second-generation inhibitor	NTKD; pan-RSK	Highly selective inhibitor with improved kinase selectivity profile; frequently used for mechanistic pathway studies	Preclinical research tool	Aronchik et al. (2014)
BIX02565	ATP-competitive small molecule	NTKD; relative selectivity for RSK2	Useful for interrogating RSK2-associated signaling biology and isoform-selective pathway dependency; limited translational development	Preclinical research tool	Romeo et al. (2012), Youn et al. (2021)
BRD7389	Multi-kinase small molecule/phenotypic screening hit	RSK pathway-associated activity; limited selectivity	Identified by functional screening; anti-proliferative activity, but less suitable as a selective mechanistic RSK inhibitor	Preclinical exploratory compound	Fomina-Yadlin et al. (2010)
BI-D1870	Reversible ATP-competitive small molecule	NTKD; pan-RSK	Widely used early tool compound; demonstrated anti-leukemic activity, mitotic disruption, and impaired AML proliferation in preclinical models	Preclinical research tool	Sapkota et al. (2007), Chae et al. (2020), Youn et al. (2021)
PMD-026	Selective ATP-competitive small molecule	NTKD; pan-RSK	Favorable early clinical tolerability, pharmacokinetics, and target engagement in solid tumors; demonstrated anti-leukemic preclinical activity in AML	Early clinical development	Beeram et al. (2021), Kong et al. (2024), Traber et al. (2026)
PMD-028	Selective RSK inhibitor	NTKD; pan-RSK	Preclinical anti-leukemic activity with continued translational development	Preclinical development	Kong et al. (2025), Traber et al. (2026)

Legend: NTKD; N-terminal kinase domain, CTKD; C-terminal kinase domain.

Taken together, RSK should be viewed less as a passive signaling relay and more as an integrative regulator of malignant cell fitness. That positioning makes it particularly attractive in myeloid malignancies, where convergent downstream dependencies may prove more therapeutically actionable than genetically diverse initiating lesions alone.

## 3 RSK signaling in myeloid malignancies

### 3.1 RSK signaling in acute myeloid leukemia

Evidence supporting a biologically relevant role for RSK signaling in AML has expanded considerably over the past several years. Early mechanistic and proteomic studies established that RSK signaling is not merely incidentally activated in leukemic cells, but may reflect a clinically meaningful signaling dependency (Chae et al., 2020; Youn et al., 2021; Kong et al., 2024). Reverse-phase protein array analysis of leukemic blasts from 483 pediatric AML patients demonstrated significantly elevated total RSK (RSK1/2/3) protein expression and increased phosphorylation of RSK at threonine 573 compared with normal CD34^+^ hematopoietic cells, supporting aberrant pathway activation in primary human disease (Chae et al., 2020). Importantly, elevated phospho-RSK was associated with shorter complete remission duration and inferior event-free survival, suggesting that activated RSK correlates with more aggressive disease biology (Chae et al., 2020). The possibility that RSK represents a true signaling dependency rather than simply a byproduct of broader oncogenic activation has gained increasing experimental support.

Pharmacologic interrogation provided some of the earliest functional evidence for RSK dependency in AML. Chae and colleagues demonstrated that treatment of AML cell lines and primary AML samples with the RSK inhibitor BI-D1870 reduced cellular viability, induced apoptosis, and disrupted cell-cycle progression (Chae et al., 2020). Mechanistically, RSK inhibition promoted accumulation of cells in G2/M and impaired metaphase-to-anaphase progression through altered spindle assembly checkpoint regulation, including disruption of CDC20 association with APC/C and increased interaction with MAD2 (Chae et al., 2020). Although BI-D1870 provided important mechanistic insight, its utility as a translational therapeutic candidate is limited by pharmacologic properties and off-target considerations common to early tool compounds. Therefore, recent work has focused on more selective strategies to define disease dependency. Analysis of public CRISPR dependency datasets has identified *RPS6KA1* (RSK1) as a selective dependency in AML, particularly within FLT3-driven disease contexts, suggesting that genetic suppression of specific RSK isoforms may impair leukemic fitness in ways that extend beyond generalized kinase inhibition (Youn et al., 2021; Kong et al., 2024; Traber et al., 2026). Taken together, current evidence supports a model in which RSK signaling contributes meaningfully to AML biology through regulation of proliferation, mitotic progression, survival, and adaptive stress responses. Whether this dependency is broadly conserved across molecular AML subtypes or enriched in specific signaling-defined disease contexts remains an important question for therapeutic development.

### 3.2 RSK signaling in FLT3-mutant AML

FLT3-mutant AML currently represents one of the clearest molecular phenotype supporting RSK dependency. Activating FLT3 mutations, particularly internal tandem duplication (FLT3-ITD) alterations, occur in a substantial subset of AML and are associated with aggressive disease behavior, increased relapse risk, and inferior clinical outcomes (Daver et al., 2019; Kazi and Ronnstrand, 2019). FLT3-ITD signaling constitutively activates multiple downstream oncogenic pathways, including STAT5, PI3K/AKT, and MAPK/ERK signaling, collectively driving leukemic proliferation, survival, and stem-like persistence (Daver et al., 2019; Kazi and Ronnstrand, 2019; Koschade et al., 2022).

RSK is especially compelling in this context because it sits directly downstream of MAPK/ERK and PI3K/PDK-1 signaling, a pathway repeatedly implicated in FLT3 inhibitor resistance (Daver et al., 2019; Kazi and Ronnstrand, 2019; Koschade et al., 2022). This downstream positioning makes RSK less vulnerable to mutation-specific escape mechanisms that frequently limit upstream kinase-directed therapy (Figure 1). RSK may also contribute directly to therapeutic resistance in FLT3-mutant AML. Resistance frequently emerges through adaptive MAPK reactivation, restoration of downstream survival signaling, and compensatory anti-apoptotic mechanisms despite continued receptor suppression (Daver et al., 2019; Kazi and Ronnstrand, 2019; Koschade et al., 2022). Resistance mechanisms include secondary kinase domain mutation, adaptive pathway rewiring, microenvironment-mediated protection, altered apoptotic signaling, and reactivation of downstream proliferative networks even in the setting of persistent FLT3 suppression (Daver et al., 2019). These observations make downstream signaling nodes particularly attractive therapeutic targets, as they may bypass genotype-specific resistance mechanisms while disrupting convergent oncogenic outputs.

RSK directly regulates survival-associated substrates including BCL2-associated agonist of cell death (BAD) and cAMP response element-binding protein (CREB) while intersecting with translational control pathways implicated in leukemic persistence (Romeo et al., 2012; Youn et al., 2021; Kong et al., 2024; Traber et al., 2026). This positioning makes RSK relevant not only to baseline oncogenic signaling, but also to resistance biology. Earlier mechanistic studies implicated RSK2 in FLT3-ITD leukemogenesis, supporting the concept that RSK family signaling contributes directly to leukemic maintenance in FLT3-driven disease (Yao et al., 2018). More recent work has strengthened this model considerably. Kong and colleagues identified RSK1 as a selective dependency in FLT3-ITD AML through integrated CRISPR dependency analysis and experimental validation, demonstrating that RSK1 suppression impaired leukemic fitness and, in some experimental contexts, produced stronger anti-leukemic effects than FLT3 suppression itself (Kong et al., 2024).

Recent translational work further supports therapeutic vulnerability in this disease setting. Selective pharmacologic inhibition with PMD-026 and PMD-028 demonstrated anti-leukemic activity in FLT3-activated AML models, suppressing cell viability, impairing clonogenic growth, and reducing leukemic burden while preserving comparatively greater normal hematopoietic function (Traber et al., 2026). Mechanistically, these studies support suppression of downstream RSK signaling outputs relevant to leukemic maintenance, reinforcing the concept that targeting signaling convergence downstream of FLT3 may offer therapeutic advantages beyond mutation-restricted kinase inhibition. Collectively, FLT3-mutant AML currently provides the strongest evidence for RSK as a therapeutically actionable signaling dependency in AML. This disease context may represent the most immediate entry point for clinical RSK-targeted strategies.

### 3.3 RSK signaling in myeloproliferative neoplasms and secondary AML

Although much of the initial focus surrounding RSK signaling in hematologic malignancy has centered on AML, more recent work suggests its relevance extends across chronic MPNs. MPNs are clonal hematopoietic disorders characterized by constitutive signaling through driver mutations involving JAK2, CALR, or MPL, resulting in persistent proliferative signaling, inflammatory cytokine production, bone marrow remodeling, and progressive stem cell dysfunction. Despite the clinical benefit of JAK inhibitor therapy, complete eradication remains limited, and progression to accelerated- or blast-phase disease remains a major clinical concern (Vainchenker and Kralovics, 2017; Bose and Verstovsek, 2020).

Given the persistent activation of MAPK/PI3K-associated signaling downstream of canonical MPN drivers, RSK represents a biologically plausible signaling vulnerability in this disease context. Recent work by Kong and colleagues demonstrated that RSK1 signifies an exploitable dependency in MPNs and secondary AML, substantially expanding the disease relevance of RSK signaling and targeting beyond *de novo* AML (Kong et al., 2025). Through integrated functional studies, the authors demonstrated that RSK1 suppression impaired malignant fitness in MPN models and reduced leukemic propagation in disease contexts associated with transformation (Kong et al., 2025). These findings suggest that RSK may represent a broader signaling vulnerability across myeloid malignancies rather than a subtype-restricted AML target. Whether RSK signaling contributes directly to leukemic transformation, inflammatory adaptation, stem cell persistence, or resistance evolution in chronic myeloid malignancies remains incompletely defined. However, current evidence strongly supports further investigation in these settings.

## 4 Therapeutic targeting of RSK

The therapeutic appeal of RSK targeting lies in its biologic position as a convergent signaling effector downstream of multiple oncogenic pathways. As shown in Figure 1, this convergence may allow therapeutic disruption of shared malignant signaling programs across molecularly distinct myeloid disease states. Unlike mutation-restricted upstream inhibitors, RSK-directed strategies may suppress common downstream survival and proliferative pathways while potentially reducing opportunities for signaling bypass through redundant receptor activation.

Early pharmacologic studies relied on first-generation RSK tool compounds that established proof-of-concept for therapeutic pathway inhibition (Table 1). BI-D1870 provided some of the earliest direct evidence of anti-leukemic activity, reducing AML viability, inducing apoptosis, and disrupting mitotic progression through altered spindle checkpoint regulation (Chae et al., 2020). Additional early compounds including SL0101, FMK, LJH685, LJI308, BIX02565, and BRD7389 further expanded mechanistic understanding of RSK kinase biology, substrate signaling, and isoform dependency across malignant systems (Cohen et al., 2005; Smith et al., 2005; Sapkota et al., 2007; Fomina-Yadlin et al., 2010; Aronchik et al., 2014; Youn et al., 2021) (Table 1). However, most remained primarily experimental tools due to limitations in kinase selectivity, pharmacokinetic properties, irreversible binding liabilities, or insufficient translational suitability (Romeo et al., 2012; Youn et al., 2021).

Subsequent efforts have focused on development of more selective, clinically tractable RSK inhibitors with improved pharmacologic properties. PMD-026 is particularly notable because it represents one of the few orally bioavailable selective RSK inhibitors to advance into human clinical development (Beeram et al., 2021). PMD-028, a related orthogonal tool compound, further supports ongoing translational optimization of RSK-directed therapeutic strategies (Traber et al., 2026). Early clinical evaluation in metastatic solid tumors demonstrated favorable tolerability, pharmacokinetic feasibility, and evidence of target engagement, providing proof-of-mechanism that sustained systemic RSK inhibition is clinically achievable (Beeram et al., 2021). Although hematologic clinical development remains limited to the recent initiation of a Phase 1 clinical trial in myelofibrosis (NCT07379125), these findings support continued evaluation of RSK-directed therapies in myeloid malignancies.

**FIGURE 2:**
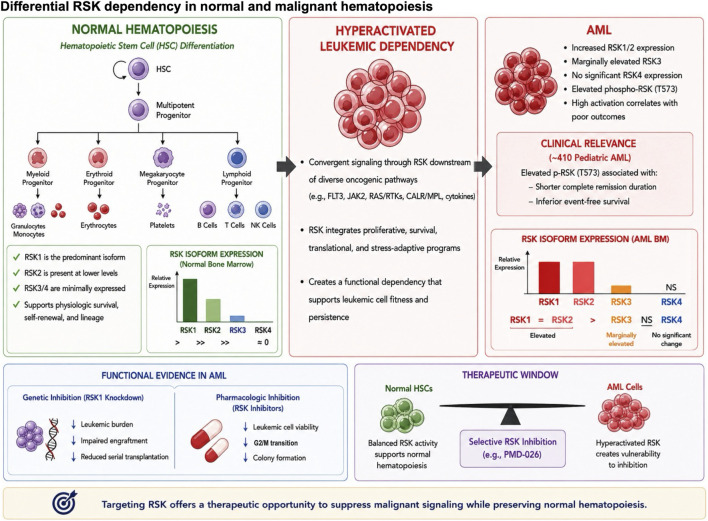
Differential RSK dependency in normal and malignant hematopoiesis. Normal hematopoietic stem and progenitor cells (HSPCs) exhibit a hierarchical pattern of RSK isoform expression, with RSK1 predominating, followed by lower RSK2 expression and minimal RSK3/4 expression, while supporting normal hematopoietic maintenance and differentiation. In contrast, AML cells demonstrate increased expression and activation of RSK family members, characterized by elevated RSK1 and RSK2 expression, modest increases in RSK3, and increased phospho-RSK signaling, whereas RSK4 expression is not significantly altered. Elevated RSK activation is associated with adverse clinical outcomes, and functional genetic and pharmacologic studies demonstrate dependence on RSK signaling for leukemic growth and survival. Together, these findings support a therapeutic window in which selective RSK inhibition may preferentially target malignant hematopoietic cells while preserving normal hematopoietic function.

More recent hematologic studies support the anti-leukemic potential of selective RSK inhibition. Pharmacologic inhibition with PMD-026 and PMD-028 demonstrated suppression of leukemic viability, clonogenic growth, and disease burden in AML models, particularly within FLT3-activated disease contexts (Traber et al., 2026). Importantly, emerging evidence suggests a therapeutic window may exist in which malignant hematopoietic dependence exceeds normal hematopoietic sensitivity, an essential consideration for kinase-directed therapies targeting pathways shared with normal stem and progenitor populations (Figure 2). Combination strategies may ultimately represent the most effective approach for RSK-directed therapy. In FLT3-mutant AML, combining FLT3 inhibition with RSK suppression may help prevent downstream pathway reactivation and resistance escape (Papaemmanuil et al., 2016; Daver et al., 2019; Kazi and Ronnstrand, 2019; Dohner et al., 2022; Koschade et al., 2022; Kong et al., 2024). In chronic myeloid neoplasms, combinations with JAK inhibitors may more effectively suppress redundant signaling architecture (Vainchenker and Kralovics, 2017; Bose and Verstovsek, 2020; Kong et al., 2025). Additional opportunities include combinations with chemotherapy such as daunomycin (Traber et al., 2026), venetoclax-based regimens, or agents targeting translational control. As RSK regulates BAD, CREB, and YB-1-associated pathways, inhibition may disrupt mechanisms implicated in venetoclax resistance (Han et al., 2020; Zhang et al., 2022; Weidenauer et al., 2023).

Although growing evidence supports therapeutic vulnerability, important translational challenges remain. Isoform specificity remains unresolved, including whether therapeutic benefit will require pan-RSK inhibition or selective targeting of dominant isoforms. The redundancy of RSK isoforms favors a pan-RSK approach. Biomarker development will also be essential because dependency on RSK signaling is unlikely to be uniform across myeloid malignancies. Compensatory signaling adaptation remains a predictable resistance mechanism that will require consideration during clinical development. Despite these challenges, the cumulative evidence supports continued translational development of RSK-targeted therapeutic strategies in myeloid malignancies.

## 5 Future perspectives

Functional dependency mapping has shifted therapeutic discovery toward convergent signaling dependencies that sustain malignant fitness across heterogeneous myeloid malignancies. RSK represents a particularly compelling example of this type of convergent signaling dependency.

Isoform specificity remains an important unresolved question. Current evidence increasingly supports RSK1 as a dominant dependency in AML and MPN contexts, whereas earlier studies implicated RSK2 (Elf et al., 2011; Kong et al., 2024; Kong et al., 2025). Clarifying isoform-specific functions will influence inhibitor design, biomarker selection, and therapeutic strategy. A pan-RSK inhibitor may be the best approach to overcome bypass via individual isoforms. It may also be a more generalizable approach to treating myeloid malignancies as well as solid tumors where RSK1 and RSK2 both serve important roles. RSK inhibition may prove most effective in combination strategies designed to suppress adaptive signaling escape. Defining the therapeutic window between malignant and normal hematopoiesis remains critical (Figure 2). Emerging evidence suggests malignant cells may exhibit greater dependence on RSK signaling than normal hematopoietic populations, while early PMD-026 clinical data support the feasibility of sustained systemic pan-RSK inhibition (Beeram et al., 2021). Although solid tumor experience does not directly predict hematologic safety, these findings support the feasibility of RSK-directed therapy. Whether this differential dependency translates into a clinically meaningful therapeutic window in myeloid malignancies remains to be determined (Traber et al., 2026). Furthermore, biomarker development remains essential as dependency on RSK signaling is unlikely to be uniform across myeloid malignancies. Identifying responsive patients will require improved understanding of pathway activation states, molecular subtype enrichment, and adaptive resistance mechanisms.

Future studies should better define how RSK contributes to leukemic persistence, inflammatory adaptation, disease progression, and isoform-specific functions in normal hematopoiesis, particularly in transformation-prone chronic myeloid malignancies. Taken together, current evidence suggests RSK has moved beyond being viewed simply as a downstream kinase and should increasingly be considered a candidate therapeutic node worthy of further clinical investigation.

## 6 Conclusion

RSK signaling has emerged as an increasingly compelling therapeutic target in myeloid malignancies. Positioned downstream of MAPK/ERK and PI3K/PDK-1 signaling, RSK serves as a convergent regulator of malignant cell fitness. Evidence from proteomic profiling, functional genetic studies, and pharmacologic interrogation supports a meaningful role for RSK signaling in AML, FLT3-mutant disease, and MPN-associated transformation. Although important translational questions remain, including isoform specificity, biomarker development, therapeutic resistance, and clinical positioning, the cumulative data support continued investigation of RSK-directed therapeutic strategies. As treatment increasingly shifts toward dependency-informed targeting, RSK represents a promising emerging vulnerability across diverse myeloid malignancies.
